# The graph structure of two-player games

**DOI:** 10.1038/s41598-023-28627-8

**Published:** 2023-02-01

**Authors:** Oliver Biggar, Iman Shames

**Affiliations:** CIICADA Lab, Australian National University, Canberra, 2601 Australia

**Keywords:** Engineering, Mathematics and computing

## Abstract

In this paper, we analyse two-player games by their *response graphs*. The response graph has nodes which are strategy profiles, with an arc between profiles if they differ in the strategy of a single player, with the direction of the arc indicating the preferred option for that player. Response graphs, and particularly their *sink strongly connected components*, play an important role in modern techniques in evolutionary game theory and multi-agent learning. We show that the response graph is a simple and well-motivated model of strategic interaction which captures many non-trivial properties of a game, despite not depending on cardinal payoffs. We characterise the games which share a response graph with a zero-sum or potential game respectively, and demonstrate a duality between these sets. This allows us to understand the influence of these properties on the response graph. The response graphs of Matching Pennies and Coordination are shown to play a key role in all two-player games: every non-iteratively-dominated strategy takes part in a subgame with these graph structures. As a corollary, any game sharing a response graph with both a zero-sum game and potential game must be dominance-solvable. Finally, we demonstrate our results on some larger games.

## Introduction

One of the most fundamental questions in game theory is that of *representing preference*^1–4^: how should we model the preferences of players over their strategies? The established solution, originating in Von Neumann and Morgenstern’s axiomatisation of *utility*^2^, is to assign to each player a *real-valued payoff*, for each combination of strategies. Soon afterwards, John Nash invented his eponymous equilibrium concept^5^, which he proved exists in every game modelled by Von Neumann–Morgenstern utility. This elegant result established the Nash equilibrium as a clear choice of the outcome of a game. Importantly, these two concepts are *mutually reinforcing*: Von Neumann–Morgenstern utility lays the mathematical foundation to prove the existence of Nash equilibria, and the existence of Nash equilibria retrospectively justifies the choice of the Von Neumann–Morgenstern model. Together, this began a flurry of game-theoretic research which cemented both Von Neumann–Morgenstern utility and the Nash equilibrium as central notions in economic thought^4^.

Unfortunately, many games do not have obvious choices of utility values. Because of this, other game models—such as *ordinal games*^6–8^—have persisted as alternatives which make weaker assumptions on what we, as modellers, must know about a strategic interaction we intend to analyse. But these models have been hindered by the dominance of the Nash equilibrium in the game theory literature^4^; without a solution concept as clear and compelling as the Nash equilibrium, such models have been unable to overtake the prevailing Von Neumann–Morgenstern approach.

However, as game theory has grown to be a significant tool in biology^9^, computer science^10^ and multi-agent learning^11^ the Nash equilibrium has been found to be a less compelling solution concept than was once thought. The first argument comes from computational complexity: Nash equilibria are intractable to compute from the description of the game^12^, even in two-player games^13^. Neither we, the analysts, nor the players themselves, can feasibly compute Nash equilibrium strategies. The second argument comes from *evolutionary game theory*, the subfield containing population dynamics and learning^14^. A series of results have established that evolution or learning rules do not^14–17^ and generally *cannot*^18,19^ converge to Nash equilibria. Instead, non-equilibrium behaviour is the rule rather than the exception, giving the Nash equilibrium relatively little predictive value^15,20–23^.

Recently, great advances have been made in AI^24–26^, using ideas from multi-agent learning^27,28^. If the Nash equilibrium is not a satisfactory notion of outcome in these fields, we are motivated to seek new approaches to evolutionary game theory which can explain and shed light on learning^16,20–22^. In particular, if our model no longer requires the Nash equilibrium, we are free to consider models which do not use Von Neumann–Morgenstern utility.

Our approach is based on *Occam’s razor*, the principle that the simplest model capable of describing the concept of interest is often the best. *We find a solution that is both computable and aligns with the outcome of evolutionary processes by simplifying our model of player preferences*.

**Table 1:** A standard presentation of the Rock–Paper–Scissors game^14^.

	Player 2		
	Rock	Paper	Scissors
Player 1			
Rock	0, 0	$-1,1$	$1,-1$
Paper	$1,-1$	0, 0	$-1,1$
Scissors	$-1,1$	$1,-1$	0, 0

**Figure 1:**
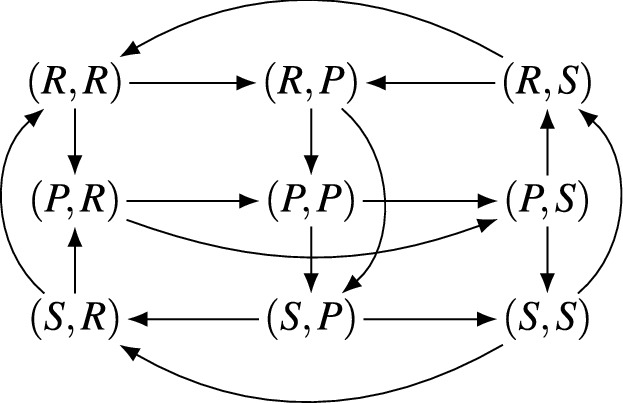
The response graph of Rock–Paper–Scissors.

**Figure 2:**
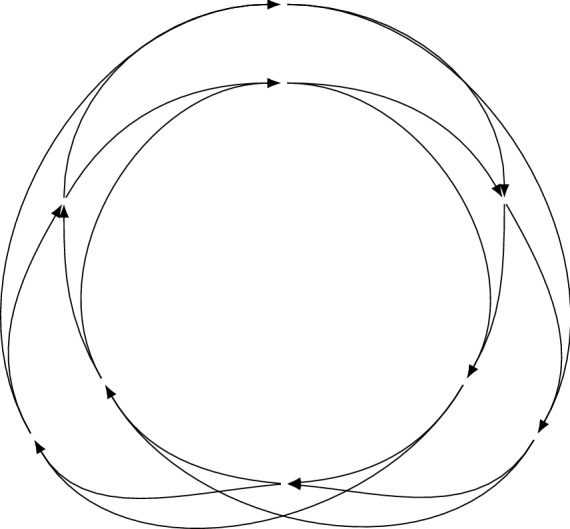
An alternate presentation of the response graph of Rock–Paper–Scissors, emphasising its Möbius strip structure.

We begin with a concrete example of a game: Rock–Paper–Scissors. In this game, each player simultaneously chooses one of ‘Rock’, ‘Paper’ or ‘Scissors’, where Rock defeats Scissors, Scissors defeats Paper, Paper defeats Rock, and playing the same option yields a tie. If we were explaining the game to another person, this description (along with the standard assumption that winning is preferred to a tie which is itself preferred to losing) is sufficient. Intuition tells us this description should also be sufficient to analyse the game mathematically. Yet, the payoffs have not been specified—this is not a game in the sense of game theory^4^. Two “Rock–Paper–Scissors” games obeying these constraints and yet differing in the payoff values are different games. We have specified only the *preference order* over each player’s strategies, given a fixed choice of strategy for the other player. For instance, if Player 1 plays Rock, we know that Player 2 prefers their options in the best-to-worst order: Paper, Rock, Scissors. This is the underlying structure of Rock–Paper–Scissors—if I reward the winner of the game with $2 instead of $1, it should not become a different game! Indeed any $$3\times 3$$ game with these preference orders, even without payoffs specified, will generally be referred to as “Rock–Paper–Scissors”. For the same reason, Rock–Paper–Scissors is often^14^ presented with the “default” 1, 0, − 1 payoffs, as in Table 1. These payoffs are serving only to instantiate the preference orders. So, can we cut out the intermediary, and define a game by the preference orders alone?

These preference orders are captured precisely in an object called the *response graph* of a game^20,29^. The nodes of this graph are the strategy profiles, and there is an arc between profiles if they differ in the strategy of a single player, with arcs directed toward the preferred profile for that player. The response graph of Rock–Paper–Scissors is shown in Fig. 1 and again in Fig. 2, with the latter emphasising the symmetric cycle structure. We can present Fig. 2 without labelling the nodes by profiles—the profiles can be reconstructed from the graph in linear time up to renaming (Theorem 3.2), so the response graph implicitly handles the problem of renaming players and strategies. Importantly for our purposes, response graphs play an important part in modern developments in machine learning and evolutionary game theory^16,20–22,30^. A key concept is the *sink strongly connected components* of the response graph (which we shall shorten to *sink components*), which are a solution concept generalising pure Nash equilibria. Recent work^31^ has shown that under the replicator dynamic, a common choice of evolutionary dynamic^14^, the sink components are contained in *sink chain components*, a topological concept which emerges from the Fundamental Theorem of Dynamical Systems^32^. Sink chain components represent the ‘long-run’ outcome of a dynamic process such as learning or evolution on a game. This result gives a compelling motivation for sink components as a dynamic—and unlike Nash equilibria, *predictive*—solution concept for games. They are also tractable to compute^20,21^. Building on these ideas, Omidshafiei et al.^21^ present a new approach called $\alpha$-*rank* for ranking the strength of agents in multi-agent settings using the response graph and sink components. When applied to a ‘biased’ Rock–Paper–Scissors game with differing payoffs in different profiles the authors find that $\alpha$-rank still gives an equal ranking to each strategy ‘Rock’, ‘Paper’ and ‘Scissors’^21^, suggesting the long-run strength of these strategies is a property of the response graph. A variant of the response graph also exists, called the *weighted response graph*, where arcs are weighted by the difference in payoff for the associated player. Weighted response graphs provide a mechanism to decompose games^29,33,34^ up to *strategic equivalence*. More recently, the spectrum^35^ of the response graph has been used to describe the topological landscape of multiplayer games^36^ for the purposes of analysing and comparing games. Sink components have also been used^37^ as an alternative measure of the Price of Anarchy^38^.

The response graph is defined by the preference orders; it does not depend closely on payoffs. If two games have different payoffs but the preference order for any given player is equal for any fixed choice of strategies for the other players, the response graphs are the same. The response graph is more general than an *ordinal game*^6,7,39^—in that model, two games are *ordinal-equivalent* if each player’s order over *all* profiles is the same. In the Rock–Paper–Scissors example, this would require modelling whether Player 1 prefers winning in the profile (Rock, Scissors) to winning in the profile (Scissors, Paper), even though they can never unilaterally choose between these profiles! The notion of *strategic equivalence*^29,34,40^ takes this into account, defining two games to be *strategically-equivalent* if the relative payoff difference between *comparable* profiles—those differing in only one player—is equal. Strategic equivalence is captured by the *weighted* response graph; it is motivated by the fact that the Nash equilibrium is invariant under strategic equivalence^29,33^. In fact, strategic equivalence is defined by the preference orders over all *mixed* profiles^41^. Unlike ordinal equivalence, strategic equivalence *does* depend on the cardinal value of payoffs. The (unweighted) response graph combines the strengths of both equivalences, generalising ordinal and strategic games into a simple and well-motivated model capturing the ‘underlying structure’ of a game^31^.

The response graph is a simple and general model. If it is to be a *good* model, by Occam’s razor, it must also be capable of describing non-trivial properties of a game. That is the goal of this paper: to establish that, despite their generality and combinatorial nature, response graphs capture important and non-trivial game-theoretic properties which extend the existing theory of two-player games. Though we focus on two-player games, we expect that the response graph approach will be equally applicable for general games. This line of inquiry allows us to conceptually separate those properties of a game which are defined by the payoffs from those which are defined by the preferences alone, and so provide a better-informed theory in applications where we cannot reliably model real-valued payoffs. Recalling the connection^20,21,31^ between the sink components and the long-run outcome of the replicator dynamics on a game, we find that investigating the sink components, as we do in this paper, sheds light on evolution and learning.

### Contributions

In this paper, we study the response graphs of two-player games. To isolate the influence of the response graph, we study games *modulo isomorphism of response graphs*. That is, we say two games are *preference-equivalent* if their response graphs are isomorphic. It is easy to see that pure Nash equilibria and strict iterated dominance of pure strategies are properties which are invariant under this equivalence relation. We define the *preference-zero-sum* and *preference-potential* game as those games which are preference-equivalent to either a zero-sum game^2^ or potential game^42^ respectively. These classes of games are particularly important in game dynamics^14^, and so understanding their sink components is a natural question of interest. Zero-sum two-player games particularly are one of the most well-studied classes of games^2^, and their definition depends crucially on payoffs. Despite this, we find that key properties of zero-sum games extend to the much broader set of preference-zero-sum games, showing that being zero-sum is to some degree a graph property. While the preference-potential games are known to be those with *acyclic* response graphs^7^, the preference-zero-sum games have—to our knowledge—never been characterised. We prove that a two-player game is preference-zero-sum if and only if it is acyclic after *reflection*, which is a reversal of preferences for one player (Corollary 4.7). Thus the graph property underlying the zero-sum property is acyclicity. We find that the existence of pure Nash equilibria in potential games extends to preference-potential games, and the uniqueness of Nash equilibria in generic zero-sum games translates to uniqueness of the sink component in generic preference-zero-sum games (Lemma 4.10).

**Figure 3:**
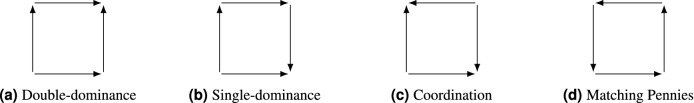
The four non-isomorphic response graphs of generic $2\times 2$ games.

The Matching Pennies and Coordination^14^ $2\times 2$ games respectively form the prototypical examples of zero-sum and potential games. Their response graphs (Fig. 3c,d) are the 4-cycle and the *reflected* 4-cycle (Definition 4.4). Remarkably, we find that these two graphs play a *fundamental role* in bringing strategic complexity to *all two-player games*. First, any two-player game which has multiple sink components must contain the response graph of CO as an induced subgraph (Theorem 4.10). Second, in any two-player game, every non-iteratively-dominated strategy takes part in a $2\times 2$ subgame whose graph is that of Matching Pennies or Coordination (Theorem 5.1). As a consequence we find a new game theory result: if all $2\times 2$ subgames of a two-player game have a dominated strategy, then the game is dominance-solvable. Combining this result with the previous characterisations, we obtain the surprising corollary that any two-player game both preference-zero-sum and preference-potential must be dominance-solvable (Corollary 5.3). These results are far-reaching, because the classes of preference-zero-sum and preference-potential games are very broad; every $2\times 2$ game is either preference-zero-sum, preference-potential, or both, in which case it is dominance-solvable. Even among $2\times 3$ games, there is *only one* generic response graph which is neither preference-zero-sum nor preference-potential (Fig. 8c).

Finally, we demonstrate our results by exploring $2\times 3$, $2\times 4$ and $3\times 3$ response graphs. We show how the techniques of the paper allow us to reason about such games easily, and we construct a stock of examples with interesting properties. For instance, we construct a generic $3\times 3$ preference-zero-sum game with a pure Nash equilibrium (Fig. 11a) and show that it is the unique response graph with these properties.

The proofs can be found in the Supplementary Material.

## Preliminaries

A *graph*^43^ is a pair $G = (N,A)$, where *N* is a finite set of *nodes* and $A\subseteq N\times N$ is a finite set of *arcs*. We depict an arc $(x,y)\in A$ by $x{\longrightarrow }y$. If for some nodes *x* and *y* we have both $(x,y)\in A$ and $(y,x)\in A$ then we refer to this pair of arcs collectively as an *undirected edge*, and depict it as ${x}-\!\!\!-\!\!\!-{y}$ . If $(x,y)\in A$ implies $(y,x)\in A$ for any pair of nodes, then all arcs are undirected edges, and we call *G* an *undirected graph*. Each graph *G* has an associated undirected graph $G'$, called the *underlying graph*, given by requiring that for each arc (*x*, *y*) in *G* there are arcs (*x*, *y*) and (*y*, *x*) in $G'$. Removing one of the arcs (*x*, *y*) or (*y*, *x*) from an undirected edge ${x}-\!\!\!-\!\!\!-{y}$ gives a standard arc, a process we call *orienting* the undirected edge. An *orientation* of an undirected graph is any graph formed by oriented some of its undirected edges. A *path* is a sequence $v_1,v_2,\dots ,v_n$ of distinct nodes where there is an arc $v_i{\longrightarrow }v_{i+1}$ for every *i* in $1,2,\dots ,n-1$. An *undirected path* is a path in the underlying graph. If there is also an arc $v_n{\longrightarrow }v_1$, we call this a *cycle*. A graph with no cycles is called *acyclic*. If there is a path from a node *v* to a node *w* we say *w is reachable from v*. Reachability defines a preorder on the nodes of a graph. Two nodes are equivalent under this preorder if both are reachable from each other. The equivalence classes of this relation are called the *strongly connected components*. The minimal elements of this order we call the *sink components*. For any subset $X\subseteq N$ of nodes, there is an associated graph given by including exactly the arcs between nodes in *X*. This is called the *subgraph induced by X* or simply an *induced subgraph*. Two graphs $(N_1,A_1)$ and $(N_2,A_2)$ are *isomorphic* if there is a map $\varphi : N_1\rightarrow N_2$ where $v{\longrightarrow }w\in A_1$ if and only if $\varphi (v){\longrightarrow }\varphi (w)\in A_2$.

All games in this paper are two-player normal-form games with finite strategy sets^4^. Such a game is defined by a pair of *payoff functions* $u_1,u_2 : S_1\times S_2 \rightarrow {\mathbb {R}}$, where $S_1$ and $S_2$ are finite sets, called the *strategy sets*, whose elements are *strategies*. If $|S_1| = n$ and $|S_2| = m$, we call the game a $n\times m$ game. A *strategy profile* is a pair $(s_1,s_2)\in S_1\times S_2$. We call $u_1(s_1,s_2)$ the *payoff* to player 1 in the profile $(s_1,s_2)$. Two profiles are *i*-*comparable* if they differ in the strategy of player *i* only, and are *comparable* if they are *i*-comparable for some *i*. We say that a strategy $s\in S_1$ *dominates* a strategy $t\in S_1$ if $u_1(s,r) > u_1(t,r)$ for every strategy $r\in S_2$, and the same definition holds analogously for player 2. The strategy *t* is called *dominated*. If we delete some dominated strategy (forming the subgame given by removing this strategy), other strategies can become dominated in the new game. This process is called *iterated elimination of dominated strategies*^4^. Any strategy deleted during this process is called *iteratively dominated*, and otherwise a strategy is said to *survive iterated dominance*. If a game has only one profile that survives iterated dominance, then that profile is the unique pure Nash equilibrium, and we call the game *dominance-solvable*. While *mixed strategies* can also dominate strategies^4^, we focus here on the case where all strategies are pure.

The *response graph* of the game is the graph whose node set is $S_1\times S_2$, with an arc $(s_1,s_2){\longrightarrow }(t_1,t_2)$ if the profiles $(s_1,s_2)$ and $(t_1,t_2)$ are *i*-comparable and $u_i(t_1,t_2) \ge u_i(s_1,s_2)$. The *weighted response graph* (called the *game graph* in^29^) has the additional property that the arc $(s_1,s_2){\longrightarrow }(t_1,t_2)$ is weighted by the non-negative number $u_i(t_1,t_2) - u_i(s_1,s_2)$. If $u_i(t_1,t_2) = u_i(s_1,s_2)$ then say player *i* is *indifferent* between $(s_1,s_2)$ and $(t_1,t_2)$, and there are arcs in both directions, that is, there is an undirected edge ${u_i(t_1,t_2)}-\!\!\!-\!\!\!-{u_i(s_1,s_2)}$. In the weighted response graph, undirected edges are weighted by zero. A *subgame* of a game is the game given by restricting $u_1$ and $u_2$ to the domain $T_1\times T_2$, where $T_1\subseteq S_1$ and $T_2\subseteq S_2$. A pure Nash equilibrium is a profile where all *i*-comparable profiles give player *i* no improvement in payoff, for any *i*. Equivalently, $(s_1,s_2)$ is a pure Nash equilibrium if and only if for every comparable profile $(t_1,t_2)$ there is an arc $(t_1,t_2){\longrightarrow }(s_1,s_2)$ in the response graph. The sink components of the response graph have also been called *Markov-Conley chains*^20,21^, but in that context they were augmented with the structure of a Markov chain.

In game theory, a property of a game is *generic* if almost all games in payoff space possess the property^44^. We shall focus one generic property in particular; specifically, the absence of undirected edges. We shall call a game *generic* if the payoffs to player *i* in two *i*-comparable profiles are never equal—that is, if its response graph has no undirected edges.

### Definition 2.1

Two two-player games are *preference-equivalent* if their response graphs are isomorphic. They are *strategically-equivalent*^29^ if their *weighted* response graphs are also isomorphic.

We observe first that strategic equivalence implies preference equivalence. Secondly, note that the graph isomorphism criterion implicitly handles renaming of strategies and reordering of players. As an example, the game $(u_1,u_2)$ and $(u_2,u_1)$ are strategically equivalent, because the map $\varphi : S_1\times S_2 \rightarrow S_2\times S_1$, $\varphi (a,b) = (b,a)$ defines an isomorphism of the weighted response graphs. While our focus is on preference-equivalence, we do make use of the more restrictive notion of strategic equivalence. Unlike preference equivalence, strategic equivalence has been well-studied in game theory^29,33,34,40,45^ because Nash equilibria are invariant under strategic equivalence^29^.

## Graphs from games

To motivate our thinking about response graphs, we begin by considering the $2\times 2$ generic games, the simplest non-trivial games. While there are infinitely many such games, there are only four non-isomorphic response graphs, which we call Matching Pennies (MP), Coordination (CO), Single-dominance (SD) and Double-dominance (DD). We can deduce this by brute force: the underlying graph of any $2\times 2$ response graph is an undirected 4-cycle, and there are four distinct orientations of this graph, shown in Fig. 3.

**Figure 4:**
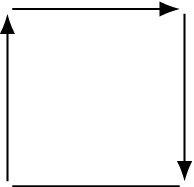
A graph that is weak MP and weak SD.

The Matching Pennies and Coordination graphs are named for some well-known games of the same name^14,30^. The Single- and Double-dominance graphs are named for the fact that they have one or two dominated strategies, respectively. These games showcase the influence of the response graph on two-player games: any game whose response graph is SD or DD is dominance-solvable; a game whose response graph is CO has two pure Nash equilibria; a game whose response graph is MP has no pure Nash equilibria. Any generic $2\times 2$ game possesses one of these response graphs. A *non-generic* $2\times 2$ game has a response graph where some of these arcs are undirected edges; an example is shown in Fig. 4. We can fit such graphs into our classification with the notion of a *weak form*.

### Definition 3.1

(*Weak form*) A graph *G* is a *weak form* of another graph *H* if *H* is an orientation of *G*.

As an example, Fig. 4 is a weak form of both SD and MP, as orienting the undirected edge gives either MP or SD. We say a graph *G* *contains* a graph *H* if *H* is an induced subgraph of *G*. We show later that weak forms of MP and CO are contained in all non-dominated two-player games. There is an important fact to note in our presentation here. Unlike in Fig. 1, where we labelled each node in the response graph by the associated profile, the graphs in Fig. 3 are not labelled by profiles. It turns out that this does not matter: if a graph is a response graph, the profiles can be recovered uniquely up to renaming of strategies.

### Theorem 3.2

*Given a graph* *G*, *we can construct a game whose response graph is* *G*, *or determine that no such games exist, in time linear in the number of arcs*.

This theorem follows from the fact that the underlying graphs of response graphs are *Hamming graphs*^46^. It tells us that the graph structure is alone sufficient to analyse the preference orders in the game. Further, this allows us to represent *implicitly* the independence of the game from renaming of strategies or reordering of players. Consequently, we can present our graphs in the natural graph-theoretic way (up to isomorphism) without losing any game-theoretic information. Consider Figs. 1 and 2, both of which depict the response graph of Rock–Paper–Scissors. While Fig. 1 mimics the payoff table structure of Table 1, Fig. 2 makes clear the symmetric Möbius-strip-like structure of the graph, which is otherwise obscure. In much the same way, we find that the presence of subgames with the structure of MP or CO can also be expressed graph-theoretically.

### Lemma 3.3

*If the response graph of a two-player game contains the response graph of a* $2\times 2$ *game, then the profiles which take part form a* $2\times 2$ *subgame*.

In particular, every appearance of MP or CO in a response graph occurs in four profiles which make up a subgame of the associated game. Hence we can interchangeably use ‘the response graph contains MP’ and ‘the game has a $2\times 2$ subgame whose response graph is isomorphic to MP’, because these statements mean the same thing.

## Two-player zero-sum and potential duality

In this section, we discuss two famous classes of games: zero-sum games^2^ and potential games^42^. We characterise these classes up to preference-equivalence; currently they have only been characterised up to the more restrictive notion of strategic equivalence^34^. Generic preference-potential games turn out to be precisely those whose response graphs are acyclic (this is straightforward to prove, and follows from results in^8^). Using a relationship between strategically-potential and strategically-zero-sum games, we establish a duality between preference-potential and preference-zero-sum games, and use this to characterise the generic preference-zero-sum games as the *reflected* acyclic games.

### Definition 4.1

A two-player game $(u_1,u_2)$ is called a *potential game*^42^ if there is a function $\phi :S_1\times S_2 \rightarrow {\mathbb {R}}$ such that for every pair of *i*-comparable profiles *p* and *q*, $\phi (p) - \phi (q) = u_i(p) - u_i(q)$. A game is *preference-potential* if it is preference-equivalent to some potential game. It is *strategically-potential* if it is strategically-equivalent to some potential game.

That is, the relative payoffs to each player can be defined by a single real-valued function, named the *potential function*, by analogy with physics. There, a dynamic *f* is called *potential* if $f = \nabla \varphi$, where $\varphi$ is a real-valued function. This means that *f* is a gradient vector field. As we know from vector calculus^47^, such vector fields are exactly those that are *conservative*. Additionally, the fundamental theorem of calculus holds, and so *f* is *path-independent*—that is, the path integral of *f* is always the difference between the values of $\varphi$ at the endpoints. In game theory, potential games are notable because they guarantee the existence of a pure Nash equilibrium^42^. Intuitively, the existence of a potential function prevents cycles of preference. This idea is well-captured by the response graph—in fact, a generic game is preference-potential if and only if its response graph is acyclic (Corollary 4.7).

### Definition 4.2

A two-player game *u* is *zero-sum* if $u_1(s_1,s_2) + u_2(s_1,s_2) = 0$ for any strategies $s_1$ and $s_2$ for players 1 and 2 respectively. A two-player game is *preference-zero-sum* if it is preference-equivalent to a zero-sum game. It is *strategically-zero-sum* if it is strategically-equivalent to a zero-sum game.

Intuitively, zero-sum games capture the notion that one player’s gain is always the other player’s loss. This model aligns closely with the recreational games from which game theory takes its name; there, if one player wins, the other must lose. From the graph perspective, this suggests that the preference orders of the players in a zero-sum game are never aligned. Hearing this, one might suspect that response graphs like CO do not occur in zero-sum games. This guess does turn out to be correct, and the insight gained leads to a characterisation of preference-zero-sum games.

### Example 4.3

**Figure 5:**
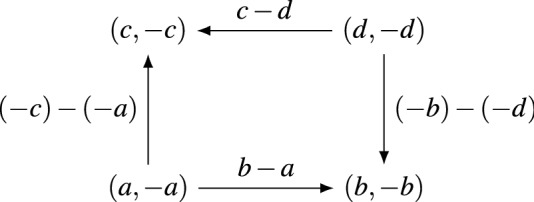
The structure of Coordination in a zero-sum game. For this to be possible, we would need $c-d > 0$, $b- a>0$, $(-b)-(-d)>0$ and $(-c)-(-a) > 0$, giving an impossible cycle $d> b> a> c > d$.

(Coordination is not preference-zero-sum) Let $(u_1,u_2)$ be a $2\times 2$ zero-sum game, with payoffs *a*, *b*, *c* and *d* for player 1 in each of the four profiles, and their negations $-a$, $-b$, $-c$ and $-d$ as the payoffs to player 2. We assume for contradiction that this game has the response graph of Coordination. The setup is shown in Fig. 5. To achieve this response graphs, the relative payoffs $c-d$, $b-a$, $a-c$ and $d-b$ must each be positive. However this implies that $c>d$, $b>a$, $a>c$ and $d>b$, giving the strict cycle $d> b> a> c > d$, which is impossible, and so we obtain a contradiction.

This example demonstrates that preference-zero-sum (and strategically-zero-sum) games are a non-trivial set of games. Usefully, the structure of the proof also suggests a way of characterising this set. The critical fact was the existence of a strict *cycle* in the underlying utilities. In some sense, zero-sum games are *acyclic*. To uncover this cycle, we use a transformation we call *reflection*.

### Definition 4.4

Let $(u_1,u_2)$ be a two-player game. The *reflected game* is $(u_1,-u_2)$. The *reversed game* is $(-u_1,-u_2)$.

Note that we made an arbitrary choice here; we could just as easily have defined the reflected game as $(-u_1,u_2)$ (‘reflecting’ the game in player 1 rather than player 2). These two games are not equivalent, but they are reversals of each other: $-(u_1,-u_2) = (-u_1,u_2)$. Our theorems are symmetric under reversal, so both choices work equally well. Reversing a game has the effect of reversing all arcs in the response graph.

### Definition 4.5

(*Path-weight*) Let $p = x_1,x_2,\dots ,x_n$ be a path in the response graph. The *path-weight* of *p* is the (signed) sum of arc labels along *p*, that is

$$\begin{aligned} {{\,\textrm{pathweight}\,}}(p) = \sum _{i=1}^{n-1} (u_{p_i}(x_i) - u_{p_i}(x_{i+1})) \end{aligned}$$

where $p_i$ is the unique player such that $x_i$ and $x_{i+1}$ are $p_i$-comparable.

We get the following theorem:

### Theorem 4.6

(Strategic zero-sum–potential duality) *A two-player game* $(u_1,u_2)$ *is strategically-potential if and only if the path-weight of any path between the same two nodes is identical. It is strategically-zero-sum if and only if its reflection* $(u_1,-u_2)$ *is strategically-potential*.

It follows easily that a potential game cannot have any strict cycles, as any path from a node to itself must have zero path-weight. Recall the analogy with path-independence and conservative vector fields in calculus. Here the path integral is replaced with the sum over weights on a path in the response graph, and one finds that the value of this ‘path integral’ is equal to the difference in potential between the two endpoints of the path. Interestingly, the reflection operation mirrors the relationship between potential and *Hamiltonian* vector fields^47,48^, which are known to be connected to zero-sum games^14,49,50^. Now we find that a combinatorial analogue of this relationship is captured in the response graph. A characterisation of preference-potential and preference-zero-sum games follows easily.

### Corollary 4.7

(Preference zero-sum–potential duality) *A two-player game* $(u_1,u_2)$ *is preference-potential if and only if every cycle in its response graph contains only undirected edges. It is preference-zero-sum if and only if its reflection* $(u_1,-u_2)$ *is preference-potential*.

**Figure 6:**
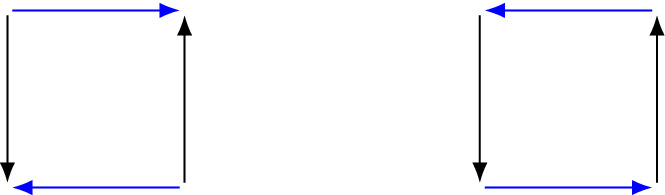
The graph of coordination (left) and its reflection in player 2, which is the graph of Matching Pennies (right). Note that reflecting the game in the preferences of player 1 swaps between the same two graphs.

It is clear now that the existence of pure Nash equilibria extends from (generic) potential games to (generic) preference-potential games. As acyclic graphs, every strongly connected component is a singleton, and so all sink components are singletons, and singleton sink components are pure Nash equilibria. It also is immediate that no generic preference-potential game ever contains MP, because this is a cycle. With this theorem in mind, we can return to Example 4.3. The reflection of Coordination is Matching Pennies—that is, a cycle—and so we conclude immediately that Coordination is not preference-zero-sum. This is shown in Fig. 6. In fact, given that the reflection of a zero-sum game cannot have any cycles, CO is never contained in any zero-sum game.

### Corollary 4.8

*Every weak form of CO contained in a preference-zero-sum game is made up of only undirected edges. Likewise, every weak form of MP contained in a preference-potential game is made up of only undirected edges.*

One of the fundamental results of two-player zero-sum games is that the set of Nash equilibria is convex. Indeed, finding Nash equilibria in a two-player game is equivalent to linear programming^4^. In non-degenerate^51^ zero-sum games, the Nash is unique, and so there is *at most one* pure Nash equilibria. Surprisingly, this uniqueness generalises to the sink components of preference-zero-sum games.

### Definition 4.9

(*Near-subgame*) Let $X \subseteq S_1\times S_2$ be a set of pairs. We say *X* is a *near-subgame* if for each pair $(s_1,s_2)$ and $(t_1,t_2)$ in *X*, at least one of $(s_1,t_2)$ or $(t_1,s_2)$ is in *X*.

If we required instead that for each $(s_1,s_2)$ and $(t_1,t_2)$ in *X*, *both* $(s_1,t_2)$ or $(t_1,s_2)$ were in *X*, then *X* is a subgame of the game. The name *near-subgame* reflects the fact that this is a slight weakening of that requirement. In Fig. 11b, we show a game whose sink component is a near-subgame but not also a subgame.

### Theorem 4.10

(Uniqueness of the sink component) *If a game does not contain coordination, then the set of sink component profiles is a near-subgame; as a consequence, the game has exactly one sink component*.

Thus we find that CO is responsible for the phenomenon of non-uniqueness of the sink components in games. Consequently it is the cause of the equilibrium selection problem^52^, at least for pure Nash equilibria. Preference-zero-sum games do not suffer from this problem, because they do not contain CO. Thus simply sharing a response graph with a zero-sum game is sufficient to ensure that there is a unique sink component.

### Corollary 4.11

*A preference-zero-sum game has exactly one sink component, and if generic has at most one pure Nash equilibrium.*

## The importance of matching pennies and coordination

In the previous section we used the $2\times 2$ games matching pennies and coordination as the prototypical examples of preference-zero-sum and preference-potential games respectively. In this section we show that these two games play a key role in introducing strategic complexity to two-player games in any number of strategies.

### Theorem 5.1

*In any non-dominance-solvable two-player game, every strategy surviving iterated dominance takes part in a subgame that is a weak form of matching pennies or coordination.*

**Figure 7:**
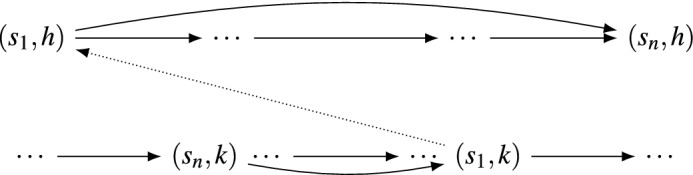
Sketch of Theorem 5.1: assuming no dominated strategies, we pick a strategy *h*, and label the other players strategies in order $s_1,\dots ,s_n$. By assumption, $s_n$ does not dominate $s_1$, so we can find other another strategy *k* where $s_1$ and $s_n$ are reversed. Pick a direction for the arc from $(s_1,h)$ to $(s_1,k)$ (the dotted arc). Requiring there be no MP or CO subgames forces all remaining arcs from $(s_i,k)$ to $(s_i,h)$ to be in the same direction; we obtain a contradiction where either *h* dominates *k* or *k* dominates *h*.

The proof is given in full the Supplementary Material. The key ideas are given in Fig. 7.

A consequence of this theorem is that *two-player games inherit dominance-solvability from their *$2\times 2$ *subgames*. That is, if every $2\times 2$ subgame has a dominated strategy, then the game is dominance-solvable. Being dominance-solvable, games without MP or CO are somewhat trivial, and so MP and CO are responsible for bringing strategic complexity to a game. In a similar way, we established above that CO brings the problem of equilibrium selection to a game. Recall Corollary 4.8: preference-zero-sum games do not contain CO, and preference-potential games do not contain MP. This theorem immediately gives us a partial converse: generic preference-zero-sum (respectively preference-potential) games either contain MP (respectively CO), or are dominance-solvable.

### Corollary 5.2

*Every strategy in a non-dominance-solvable preference-zero-sum game takes part in an MP subgame. Likewise, every strategy in a non-dominance-solvable preference-potential game takes part in an CO subgame.*

Matching Pennies is truly the prototypical preference-zero-sum response graph—not only is it the simplest example of such, but all non-dominance-solvable preference-zero-sum games contain it. The same is true for Coordination and two-player preference-potential games. As a consequence, we find that the intersection of games which are both generic preference-zero-sum and generic preference-potential can contain neither MP nor CO, and thus must be dominance-solvable. This ties together our characterisations of preference-potential and preference-zero-sum games and connects them to the concept of iterated dominance.

### Corollary 5.3

(Zero-sum–potential–dominance theorem) *Any generic game that is both preference-zero-sum and preference-potential is dominance-solvable.*

To demonstrate this result, consider the generic $2\times 2$ games. As acyclic graphs, DD, SD and CO are preference-potential. As reflected acyclic graphs, DD, SD and MP are preference-zero-sum (Corollary 4.7). As both preference-potential and preference-zero-sum games, DD and SD are dominance-solvable. Thus *every* generic $2\times 2$ game is either preference-potential, preference-zero-sum, or dominance-solvable.

This highlights another important point: the sets of preference-zero-sum games and preference-potential games are quite broad, much more so than zero-sum or even strategically zero-sum games. In a similar result^34^, the authors proved that any two-player game both strategically-potential and strategically-zero-sum must have a *dominant strategy* for each player. While this is an interesting result, its scope is more limited than Corollary 5.3; any game preference-equivalent to both a zero-sum and potential game is certainly strategically equivalent to both, but the converse does not hold. For instance, no game with the response graph of SD can ever be strategically-potential and strategically-zero-sum (there are no weights such that the graph and its reflection are both have the same path-weights on all paths between the same profiles) but SD is preference-zero-sum and preference-potential and so falls under the wider purview of our theorem. There are even cases^53^ of the explicit study of the yet-more-restricted case of games that are both zero-sum and potential, without it being noted that these games are all dominance-solvable.

## Applications

The generic $2\times 2$ games (Fig. 3) have served as useful examples throughout this paper. This is particularly true of MP and CO, the two without dominated strategies, which also served as our prototypical examples of preference-zero-sum and preference-potential games. However, not all interesting properties of two-player games can be captured in just these graphs. In this section, we discuss how the properties of response graph, particularly being preference-zero-sum and preference-potential, extend to larger two-player games, such as $2\times 3$, $2\times 4$ and $3\times 3$. It is our goal to build the reader’s intuition about response graphs and to provide a stock of example graphs with interesting game-theoretic properties. We also intend to demonstrate how the theorems of the paper can help us to analyse games. To keep things simple, we will focus on games without dominated strategies. Games which possess dominated strategies can be simplified into smaller games by deleting those strategies^4^. By Corollary 5.3, these graphs split into three categories: preference-zero-sum only, preference-potential only, and neither—games which are both preference-zero-sum and preference-potential always have a dominated strategy.

**Figure 8:**
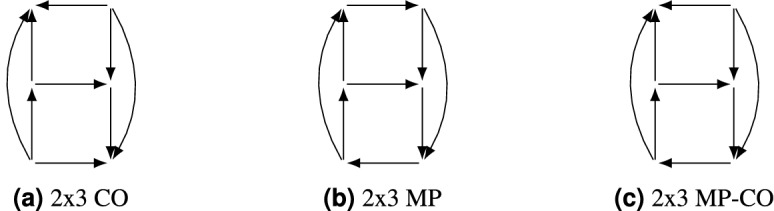
The three response graphs of generic non-dominated $2\times 3$ games.

**Figure 9:**
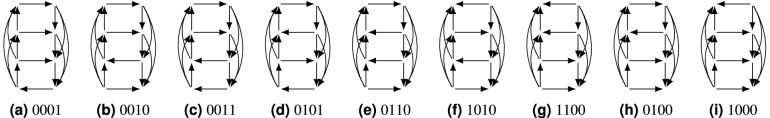
The nine response graphs of generic non-dominated $2\times 4$ games. The binary encoding defines the direction of each arc for player 2. (**a**,**c**) preference-zero-sum, and (**g**,**i**) preference-potential.

We begin with the generic $2\times 3$ games. There are exactly three such graphs without dominated strategies, as the following argument shows: in order for there to be no dominated strategies, the three-strategy player (we assume player 1) must prefer their strategies in opposite orders for each of the two strategies of player 2. It remains only to choose player 2’s preferences for each choice of strategy for player 1, which leads to the three graphs shown in Fig. 8. We can distinguish these graphs via their $2\times 2$ subgames: Fig. 8b contains two MP subgames and a SD subgame, so is preference-zero-sum; Fig. 8a contains two CO subgames and a SD subgame, so is preference-potential, and in fact is the reflection of Fig. 8b; Fig. 8c contains MP, CO and SD subgames and so is neither preference-potential nor preference-zero-sum, and so is the *unique minimal example* of such a graph. We call these ‘$2\times 3$ MP’, ‘$2\times 3$ CO’ and ‘$2\times 3$ MP-CO’ by analogy with the $2\times 2$ case. All of these graphs are isomorphic to themselves under reversal, and the $2\times 3$ MP-CO game is also isomorphic to itself after reflection of either player. A similar argument also works to classify the $2\times 4$ games. In that case there are 9 distinct response graphs with no dominated strategies (Fig. 9): two are preference-zero-sum (Fig. 9a,c), two preference-potential (Fig. 9g,i), and five neither.

**Figure 10:**
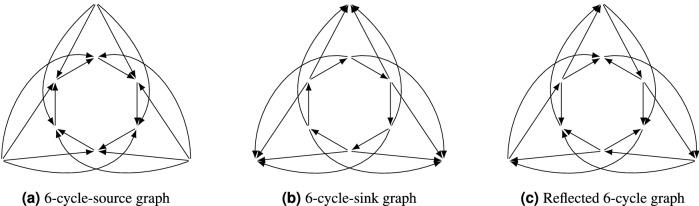
The 6-cycle-source graph (**a**) and its reversal, the 6-cycle-sink graph (**b**), do not contain MP and yet are not preference-potential. The reflection of either game in either player gives the Reflected 6-cycle graph (**c**), which is the unique $3\times 3$ game which does not contain CO yet is not preference-zero-sum.

There are 156 distinct response graphs of $3\times 3$ generic games without dominated strategies, which can be found by a computer search. Of these, 25 are preference-zero-sum and 30 are preference-potential, and the remaining 101 are neither (Corollary 5.3). We will now discuss a few of these which are useful examples of particular game-theoretic properties. In generic $2\times 2$ and $2\times 3$ games, any game without an MP was acyclic and thus preference-potential. In generic $3\times 3$ this becomes no longer true, and there are exactly two graphs (Fig. 10a,b) which do not contain a 4-cycle (an MP) and yet do contain a 6-cycle (it is easy to see that every $3\times 3$ game which has a 5-cycle must have a 4-cycle). One way to see this is by applying Theorem 5.1, using the following argument: suppose our $3\times 3$ game has six profiles which take part in a cycle, and no smaller cycle. By Theorem 5.1, each strategy must participate in a CO subgame, as we have assumed there are no MP subgames. One finds only two possibilities: either the three remaining nodes are all sources with arcs into the 6-cycle (Fig. 10a), or they are all sinks with arcs from the 6-cycle (Fig. 10b). We call these graphs the 6-cycle-source and -sink graphs respectively. By the same reasoning, the reflection of either of these graphs in either player must give a graph which has no CO subgame and yet is not preference-zero-sum. All choices of reflection are in fact isomorphic, giving a graph which we call the Reflected 6-cycle graph (Fig. 10c). This is the smallest example of a generic $3\times 3$ game without dominated strategies which does not contain CO but is also not preference-zero-sum. Its reversal is itself.

**Figure 11:**
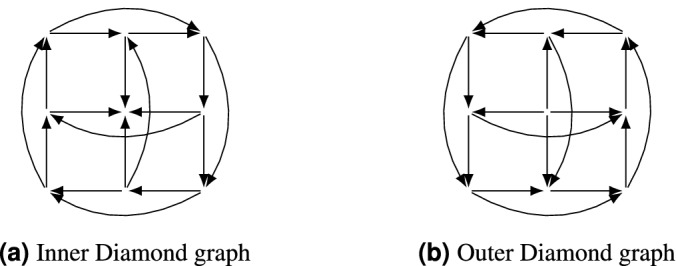
The inner and outer diamond graphs.

Corollaries 5.2 and  5.3 together tell us that in preference-zero-sum games without dominated strategies, every strategy must take part in a MP subgame. This leads to response graphs which are typically highly connected, like MP itself. However, in $3\times 3$ games we find the first examples of preference-zero-sum games which are *not* strongly connected and yet have no dominated strategies. We call these the Inner and Outer Diamond graphs, for their shape (Fig. 11a,b). They are reversals of each other. These two graphs are useful examples: the Inner Diamond graph is the smallest game demonstrating that generic preference-zero-sum games can have pure Nash equilibria. The Outer Diamond game is the smallest example of a generic preference-zero-sum game whose sink component is not a subgame (though it is a *near-subgame*, by Theorem 4.10).

## Conclusions

In this paper, we discussed the response graphs of two-player games. The response graph is a model of game which captures only the underlying notion of strategic preference and not the cardinal values for payoffs, in other words, a model that does not concern itself with the actual payoff values and only focuses on the ordering of the payoffs. This allows response graphs to be used as a model in circumstances when access to or knowledge of cardinal payoffs is implausible. The notion of *preferences* agrees with our intuitive notions about simple games such as Rock–Paper–Scissors. While many key game-theoretic concepts—such as dominance and pure Nash equilibria—depend only on which strategies are preferred, the response graph has received little direct study and few of its general properties are known. In this paper, we demonstrated that the response graph contains significant mathematical structure, and its study leads to new game-theoretic insight. We showed first that two-player potential and zero-sum games, two of the best-studied classes of game, have very natural characterisations in terms of response graph structure—specifically, acyclicity. Furthermore, we established that the key equilibrium properties of these games translate to analogous properties of the sink component of the response graph. We then argued that the response graphs of the Matching Pennies and Coordination games play a key role in two-player games: any game not including these games as subgames must be dominance-solvable. In summary, we found, and strove to convey the message, that the response graph is an interesting game-theoretic object which we believe merits further study.

## Supplementary Information

Supplementary Information.

## Data Availability

The contributions of this paper are mathematical, so no data was used. The proofs can be found in the Supplementary Material.
