# Supplementary material for: The graph structure of two-player games
Source: Sci Rep. 2023 Feb 1;13:1833. doi: 10.1038/s41598-023-28627-8 (PMC9892046; doi:10.1038/s41598-023-28627-8)
Supplement: Supplementary file 1 — Supplementary Information. [file 41598_2023_28627_MOESM1_ESM.pdf]

The graph structure of two-player games: supplementary material

Oliver Biggar[1] **and Iman Shames**[1]

[1]CIICADA Lab, Australian National University, Canberra, 2601, Australia
[*]oliver.biggar@anu.edu.au

ABSTRACT

This document contains proofs and technical discussion omitted from the main manuscript.

1 Preliminaries

A *graph*[1] is a pair $G = (N, A)$, where N is a finite set of *nodes* and $A \subseteq N \times N$ is a finite set of *arcs*. We depict an arc $(x, y) \in A$ by $x \longrightarrow y$. If for some nodes x and y we have both $(x, y) \in A$ and $(y, x) \in A$ then we refer to this pair of arcs collectively as an *undirected edge*, and depict it as $x \;\text{———}\; y$. If $(x, y) \in A$ implies $(y, x) \in A$ for any pair of nodes, then all arcs are undirected edges, and we call G an *undirected graph*. Each graph G has an associated undirected graph G', called the *underlying graph*, given by requiring that for each arc (x, y) in G there are arcs (x, y) and (y, x) in G'. Removing one of the arcs (x, y) or (y, x) from an undirected edge $x \;\text{———}\; y$ gives a standard arc, a process we call *orienting* the undirected edge. An *orientation* of an undirected graph is any graph formed by oriented some of its undirected edges. A *path* is a sequence $v_1, v_2, \ldots, v_n$ of distinct nodes where there is an arc $v_i \longrightarrow v_{i+1}$ for every i in $1, 2, \ldots, n-1$. An *undirected path* is a path in the underlying graph. If there is also an arc $v_n \longrightarrow v_1$, we call this a *cycle*. A graph with no cycles is called *acyclic*. If there is a path from a node v to a node w we say *w is reachable from v*. Reachability defines a preorder on the nodes of a graph. Two nodes are equivalent under this preorder if both are reachable from each other. The equivalence classes of this relation are called the *strongly connected components*. The minimal elements of this order we call the *sink components*. For any subset $X \subseteq N$ of nodes, there is an associated graph given by including exactly the arcs between nodes in X. This is called the *subgraph induced by X* or simply an *induced subgraph*. Two graphs (N_1, A_1) and (N_2, A_2) are *isomorphic* if there is a map $\varphi : N_1 \to N_2$ where $v \longrightarrow w \in A_1$ if and only if $\varphi(v) \longrightarrow \varphi(w) \in A_2$.

All games in this paper are two-player normal-form games with finite strategy sets[2]. Such a game is defined by a pair of *payoff functions* $u_1, u_2 : S_1 \times S_2 \to \mathbb{R}$, where S_1 and S_2 are finite sets, called the *strategy sets*, whose elements are *strategies*. If $|S_1| = n$ and $|S_2| = m$, we call the game a $n \times m$ game. A *strategy profile* is a pair $(s_1, s_2) \in S_1 \times S_2$. We call $u_1(s_1, s_2)$ the *payoff* to player 1 in the profile (s_1, s_2). Two profiles are *i-comparable* if they differ in the strategy of player i only, and are *comparable* if they are i-comparable for some i. We say that a strategy $s \in S_1$ *dominates* a strategy $t \in S_1$ if $u_1(s, r) > u_1(t, r)$ for every strategy $r \in S_2$, and the same definition holds analogously for player 2. The strategy t is called *dominated*. If we delete some dominated strategy (forming the subgame given by removing this strategy), other strategies can become dominated in the new game. This process is called *iterated elimination of dominated strategies*[2]. Any strategy deleted during this process is called *iteratively dominated*, and otherwise a strategy is said to *survive iterated dominance*. If a game has only one profile that survives iterated dominance, then that profile is the unique pure Nash equilibrium, and we call the game *dominance-solvable*. While *mixed strategies* can also dominate strategies[2], we focus here on the case where all strategies are pure.

The *response graph* of the game is the graph whose node set is $S_1 \times S_2$, with an arc $(s_1, s_2) \longrightarrow (t_1, t_2)$ if the profiles (s_1, s_2) and (t_1, t_2) are i-comparable and $u_i(t_1, t_2) \geq u_i(s_1, s_2)$. The *weighted response graph* (called the *game graph* in[3]) has the additional property that the arc $(s_1, s_2) \longrightarrow (t_1, t_2)$ is weighted by the non-negative number $u_i(t_1, t_2) - u_i(s_1, s_2)$. If $u_i(t_1, t_2) = u_i(s_1, s_2)$ then say player i is *indifferent* between (s_1, s_2) and (t_1, t_2), and there are arcs in both directions, that is, there is an undirected edge $u_i(t_1, t_2) \;\text{———}\; u_i(s_1, s_2)$. In the weighted response graph, undirected edges are weighted by zero. A *subgame* of a game is the game given by restricting u_1 and u_2 to the domain $T_1 \times T_2$, where $T_1 \subseteq S_1$ and $T_2 \subseteq S_2$. A pure

Nash equilibrium is a profile where all i-comparable profiles give player i no improvement in payoff, for any i. Equivalently, (s_1,s_2) is a pure Nash equilibrium if and only if for every comparable profile (t_1,t_2) there is an arc $(t_1,t_2) \longrightarrow (s_1,s_2)$ in the response graph. The sink components of the response graph have also been called *Markov-Conley chains*[4,5], but in that context they were augmented with the structure of a Markov chain.

In game theory, a property of a game is *generic* if almost all games in payoff space possess the property[6]. We shall focus one generic property in particular; specifically, the absence of undirected edges. We shall call a game *generic* if the payoffs to player i in two i-comparable profiles are never equal—that is, if its response graph has no undirected edges.

Definition 1.1. Two two-player games are *preference-equivalent* if their response graphs are isomorphic. They are *strategically-equivalent*[3] if their *weighted* response graphs are also isomorphic.

We observe first that strategic equivalence implies preference equivalence. Secondly, note that the graph isomorphism criterion implicitly handles renaming of strategies and reordering of players. As an example, the game (u_1,u_2) and (u_2,u_1) are strategically equivalent, because the map $\varphi : S_1 \times S_2 \to S_2 \times S_1$, $\varphi(a,b) = (b,a)$ defines an isomorphism of the weighted response graphs. While our focus is on preference-equivalence, we do make use of the more restrictive notion of strategic equivalence. Unlike preference equivalence, strategic equivalence has been well-studied in game theory[3,7–10] because Nash equilibria are invariant under strategic equivalence[3].

2 Graphs from Games

Definition 2.1 (Weak Form)**.** A graph G is a *weak form* of another graph H if H is an orientation of G.

Theorem 2.2. *Given a graph G, we can construct a game whose response graph is G, or determine that no such games exist, in time linear in the number of arcs.*

Proof. In [11, Theorem 22.2] it is established that Hamming graphs, the underlying graphs of response graphs, can be recognised and given a labelling by tuples in $S_1 \times S_2 \times \cdots \times S_N$ such that two adjacent nodes differ in a single entry of their tuples. This can be done in time $O(m)$ where m is the number of edges. This labelling is unique up to the choice of sets S_i.

Let G be a given graph. To check if G is the response graph of a game, we first check if its underlying graph is a Hamming graph, using the above technique. If so, its node set is $S_1 \times S_2 \times \cdots \times S_n$, and we assign these sets as the strategy sets for each player. It remains only to check that the graph is oriented such that, for each fixed choice of strategies for $N-1$ players, the strategy profiles for each choice of the remaining player are totally ordered. We can do this by iteration; for each player i, iterate through each combination of strategies for the other players and verify that the associated subgraph is directed such that it is acyclic. If not, we reject the graph. This loop examines each edge of the graph once, so is $O(m)$.

Now we show that any graph G satisfying these criteria is indeed a response graph. Fix some player i, with $|S_i| = k$. For each fixed choice of strategy s_{-i} to all players other than i, the associated subgraph of the response graph is a total order. We assign the payoffs $1,2,\ldots,k$ to the strategies in S_i, in this order. The result is a game whose response graph is G. □

Lemma 2.3. If the response graph of a two-player game contains the response graph of a 2×2 game, then the profiles which take part form a 2×2 subgame.

Proof. The result is a special case of a general property of Hamming graphs.

Suppose p_1,p_2,p_3,p_4 are profiles, and the underlying graph of the subgame induced by them is a 4-cycle, with nodes in this order. Let $p_1 = (a_1,b_1)$ and $p_2 = (a_2,b_1)$ without loss of generality. Then p_3 is comparable to p_2 but not p_1, so again without loss of generality $p_3 = (a_2,b_2)$. Finally, p_4 is comparable to p_1 and p_3 but not p_2, and so we conclude that $p_4 = (a_1,b_2)$. Thus these profiles correspond to the subgame $\{a_1,a_2\} \times \{b_1,b_2\}$. □

3 Two-Player Zero-Sum and Potential Duality

Definition 3.1. A two-player game (u_1,u_2) is called a *potential game*[12] if there is a function $\phi : S_1 \times S_2 \to \mathbb{R}$ such that for every pair of i-comparable profiles p and q, $\phi(p) - \phi(q) = u_i(p) - u_i(q)$. A game is *preference-potential* if it is preference-equivalent to some potential game. It is *strategically-potential* if it is strategically-equivalent to some potential game.

Definition 3.2. A two-player game u is *zero-sum* if $u_1(s_1,s_2) + u_2(s_1,s_2) = 0$ for any strategies s_1 and s_2 for players 1 and 2 respectively. A two-player game is *preference-zero-sum* if it is preference-equivalent to a zero-sum game. It is *strategically-zero-sum* if it is strategically-equivalent to a zero-sum game.

Definition 3.3. Let (u_1, u_2) be a two-player game. The *reflected game* is $(u_1, -u_2)$. The *reversed game* is $(-u_1, -u_2)$.

Definition 3.4 (Path-weight)**.** Let $p = x_1, x_2, \ldots, x_n$ be a path in the response graph. The *path-weight* of p is the (signed) sum of arc labels along p, that is

$$\text{pathweight}(p) = \sum_{i=1}^{n-1} (u_{p_i}(x_i) - u_{p_i}(x_{i+1}))$$

where p_i is the unique player such that x_i and x_{i+1} are p_i-comparable.

Theorem 3.5 (Strategic Zero-Sum–Potential Duality)**.** *A two-player game (u_1, u_2) is strategically-potential if and only if the path-weight of any path between the same two nodes is identical. It is strategically-zero-sum if and only if its reflection $(u_1, -u_2)$ is strategically-potential.*

Proof. Claim: A game (u_1, u_2) is strategically-potential if and only if all undirected paths between any two profiles x_1 and x_n have the same path-weight.

Suppose (u_1, u_2) is potential with potential function ϕ, and let $p_1 = x_1, x_2, \ldots, x_n$ and $p_2 = x_1, y_1, \ldots, y_m, x_n$ be two paths between profiles x_1 and x_2. The path-weight is

$$\text{pathweight}(p_1) = \sum_{i=1}^{n-1} (u_{p_i}(x_i) - u_{p_i}(x_{i+1}))$$

$$\text{pathweight}(p_1) = \sum_{i=1}^{n-1} (\phi(x_i) - \phi(x_{i+1})) \qquad \text{(the game is potential)}$$

$$\text{pathweight}(p_1) = \phi(x_1) - \phi(x_n)$$

where we have used the fact that the sum is telescoping. By identical reasoning, $\text{pathweight}(p_2) = \phi(x_1) - \phi(x_2) = \text{pathweight}(p_1)$.

For the converse, suppose that in (u_1, u_2) the path-weight on any path between the same two nodes is equal. Define an order $v \preceq w$ if the path-weight of any path from v to w is non-negative. This is well-defined because all such paths have the same path-weight. This is reflexive, and we can see that it is transitive by the following. If $v \preceq w \preceq t$, then the path-weight from v to t is the sum of the path-weights of paths from v to w and w to t respectively, and these are each non-negative, so the path-weight from v to t is also non-negative.Since the underlying graph is connected, this order is *total*. This order must have minimal elements as it is finite. Choose one, call it z. Define a potential function ϕ as follows. Set $\phi(z) = 0$, and for any other node x, define $\phi(x) = \text{pathweight}(p_{x \to z})$, where $p_{x \to z}$ is any undirected path from x to z.

Now we show this is indeed a potential function. Let v and w be i-comparable profiles. Choose paths $p_{v \to z}$ and $p_{z \to w}$. Since u is path-independent, the path-weight along the one-step path $p = u, v$, which is $u_i(v) - u_i(w)$, must be equal to the path-weight of the concatenated path $p_{u \to z} p_{z \to v}$, and this is equal to $\phi(u) + (-\phi(v))$, so ϕ is a potential function. This establishes the claim.

Observe that (u_1, u_2) and (v_1, v_2) are strategically equivalent if and only if $(u_1, -u_2)$ and $(v_1, -v_2)$ are strategically equivalent. Suppose (u_1, u_2) is zero-sum. Then the payoff in any profile (s_i, s_j) is $(x_{i,j}, -x_{i,j})$ for some real $x_{i,j}$. In the reflected game $(u_1, -u_2)$ the payoff is $(x_{i,j}, x_{i,j})$. This game is an *identical interest game*, and thus a potential game, with potential function $\phi : S_1 \times S_2 \to \mathbb{R}$, $\phi(s_i, s_j) = x_{i,j}$. Thus if (v_1, v_2) is strategically-equivalent to (u_1, u_2), then $(v_1, -v_2)$ is strategically-equivalent to $(u_1, -u_2)$, so is strategically-potential. For the converse, suppose that (u_1, u_2) is potential, with potential function $\phi : S_1 \times S_2 \to \mathbb{R}$. Then the game $(\phi, -\phi)$ is clearly a zero-sum game, and its reflection (ϕ, ϕ) is a potential game with potential ϕ by the above. For either player i and i-comparable profiles v and w, $u_i(v) - u_i(w) = \phi(v) - \phi(w)$ and so (u_1, u_2) is strategically equivalent to (ϕ, ϕ). By transitivity, any game strategically equivalent to (u_1, u_2) has a reflection which is strategically-zero-sum, as it is strategically equivalent to $(\phi, -\phi)$. ☐

Corollary 3.6 (Preference Zero-Sum–Potential Duality)**.** A two-player game (u_1, u_2) is preference-potential if and only if every cycle in its response graph contains only undirected edges. It is preference-zero-sum if and only if its reflection $(u_1, -u_2)$ is preference-potential.

Proof. If the game is potential with potential function ϕ, then for any cycle $x_1, \ldots, x_n, x_1$ we have $\phi(x_1) \leq \phi(x_2) \leq \cdots \leq \phi(x_n) \leq \phi(x_1)$, which implies $\phi(x_1) = \phi(x_2) = \cdots = \phi(x_n)$. Thus the cycle contains only undirected edges.

For the converse, we will use an approach similar to Theorem 3.5, where we will construct a potential function and argue that the associated potential game has this response graph. Suppose that in (u_1,u_2) every cycle contains only undirected edges. Define an order $v \preceq w$ if there is a directed path from v to w. This is the reachability partial order of the graph. We can always assign real numbers r_i to each strongly connected component s_i such that $r_i < r_j$ if $s_i \prec s_j$. Choose some such values, and then define $\phi(v) = r_i$, where v is in the connected component s_i.

To see that this is a potential function with the same response graph, let v and w be i-comparable profiles. If the arc between v and w is an undirected edge, then $v \preceq w$ and $w \preceq v$ so they are in the same connected component and $\phi(v) = \phi(w)$. Conversely, if $\phi(v) = \phi(w)$ then $v \preceq w$ and $w \preceq v$, and so there is a cycle from v to itself containing w; by the above, all arcs on this cycle are undirected edges, so the arc between v and w is an undirected edge. If the arc is not an undirected edge, so $v \longrightarrow w$, then $v \prec w$ and by construction $\phi(v) < \phi(w)$. Conversely, if $\phi(v) < \phi(w)$ then v and w are in different connected components, and the component of v precedes that of w in the reachability order, so there is an arc $v \longrightarrow w$. Hence ϕ defines a potential game with the same response graph as (u_1,u_2).

Now suppose (u_1,u_2) is preference-zero-sum, so preference-equivalent to some zero-sum game (z_1,z_2). By Theorem 3.5, $(z_1,-z_2)$ is potential, and $(u_1,-u_2)$ is preference-equivalent to $(z_1,-z_2)$. For the converse, suppose $(u_1,-u_2)$ is preference-equivalent to some potential game (w_1,w_2). By Theorem 3.5, $(w_1,-w_2)$ is strategically equivalent to a zero-sum game (z_1,z_2). By transitivity, and the fact that strategically equivalent games are preference-equivalent, (u_1,u_2) is preference-equivalent to (z_1,z_2). □

Corollary 3.7. Every weak form of CO contained in a preference-zero-sum game is made up of only undirected edges. Likewise, every weak form of MP contained in a preference-potential game is made up of only undirected edges.

Proof. If a game contains a weak form of CO then it is a subgame (Lemma 2.3). Any such subgame becomes a subgame isomorphic to a weak form of MP in the reflected game. However, in a preference-potential game, any MP subgame must consist of only undirected edges, and so in a preference-zero-sum game any CO must consist of only undirected edges, by Theorem 3.5. □

Definition 3.8. Let $X \subseteq S_1 \times S_2$ be a set of pairs. We say X is a *near-subgame* if for each pair (s_1,s_2) and (t_1,t_2) in X, at least one of (s_1,t_2) or (t_1,s_2) is in X.

Theorem 3.9 (Uniqueness of the sink component)**.** *If a game does not contain Coordination, then the set of sink component profiles is a near-subgame; as a consequence, the game has exactly one sink component.*

Proof. Let (a,b) and (x,y) be profiles contained in (possibly different) sink components. For contradiction, assume that neither (a,y) nor (x,b) are in sink components, and so neither (a,b) nor (x,y) have arcs to them. However, we find that the subgame $\{a,x\} \times \{b,y\}$ is the response graph of CO, which contradicts our assumption. Thus the sink component profiles are a near-subgame, and without loss of generality there is an arc $(a,b) \longrightarrow (x,b)$. Now we show uniqueness by demonstrating that (a,b) and (x,y) must be in the same sink component. Strongly connected components define an equivalence relation on nodes, so we need only show that there is a node in common. Since there is an arc $(a,b) \longrightarrow (x,b)$, (x,b) is in the same sink component as (a,b). If there is an arc $(x,b) \longrightarrow (x,y)$ then (x,y) is also in this component; if there is an arc $(x,y) \longrightarrow (x,b)$ then (x,y) is in the same sink component as (x,b) and so, by transitivity, (a,b). As at least one of these arcs must exist, the result is proved. □

Corollary 3.10. A preference-zero-sum game has exactly one sink component, and if generic has at most one pure NE.

Proof. Preference-zero-sum games do not contain CO (Corollary 3.7), and so there is one sink component by Theorem 3.9. Pure Nash equilibrium are singleton sink components. As there is exactly one sink component, this is the only pure NE. □

4 The Importance of Matching Pennies and Coordination

Theorem 4.1. *In any non-dominance-solvable two-player game, every strategy surviving iterated dominance takes part in a subgame that is a weak form of Matching Pennies or Coordination.*

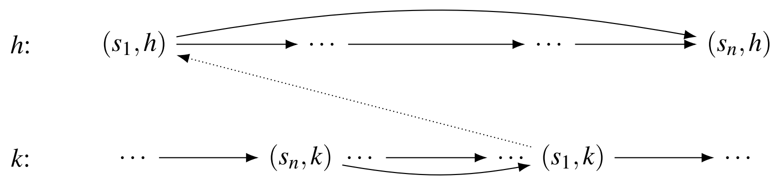

Figure 1. The initial setup of the proof, with the direction of the dotted arc from k to h by assumption of case (2).

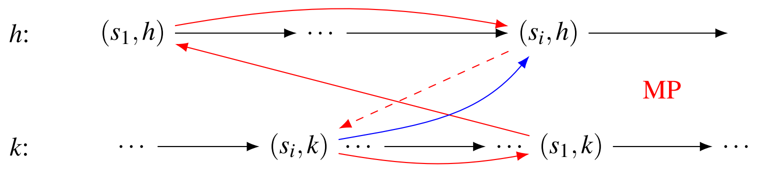

Figure 2. For s_i where (s_i,k) precedes (s_1,k), the arc $(s_i,k) \longrightarrow (s_i,h)$ must go from k to h (blue); if instead the arc goes from h to k (red, dashed) the subgame $\{s_i,s_1\} \times \{h,k\}$ is MP.

Proof. We assume for contradiction that all iteratively dominated strategies have been removed. As the game is not dominance-solvable, there are at least two strategies remaining for both players and no strategy dominates another. Now let h be some strategy for player 2 (without loss of generality), and suppose that h never takes part in a subgame isomorphic to a weak form of MP or CO. We will demonstrate a contradiction by showing that a dominated strategy must exist. Let $s_1,\ldots,s_n$ be the strategies for player 1, ordered by payoff for player 1 when player 2 plays h, that is, $u_1(s_1,h) \le u_1(s_2,h) \le \cdots \le u_1(s_n,h)$. This is pictorially shown in Figure 1.

Suppose first that $u_1(s_1,h) = u_1(s_n,h)$, then $u_1(s_i,h) = u_1(s_j,h)$ for any s_i and s_j. Pick any other strategy k for player 2. If there exists s_i and s_j where $u_2(s_i,h) \le u_2(s_i,k)$ and $u_2(s_j,h) \ge u_2(s_j,k)$, then $\{s_i,s_j\} \times \{h,k\}$ is a weak form of MP or CO. If there do not exist such a pair of s_i and s_j, then we find that $u_2(s_i,h) < u_2(s_i,k)$ for every s_i or $u_2(s_i,h) > u_2(s_i,k)$ for every s_i, implying that h dominates or is dominated by k, contradicting our assumption.

Now suppose that $u_1(s_1,h) < u_1(s_n,h)$. No strategy dominates any other, so s_n does not dominate s_1, and thus there is some strategy k for player 2 where $u_1(s_n,k) \le u_1(s_1,k)$. If $u_1(s_1,h) = u_1(s_1,k)$, then the subgame $\{s_1,s_n\} \times \{h,k\}$ is a weak form of MP or CO. Assuming this does not hold, there are two cases, for which the argument is symmetric while only reversing the role of h and k and MP and CO. In case (1), player 2 prefers h when player 1 plays s_1, so $u_2(s_1,h) > u_2(s_1,k)$, and case (2) is the opposite, where $u_2(s_1,h) < u_2(s_1,k)$. Case (1) is depicted in Figure 1.

Now let s_i be any strategy with $u_1(s_i,k) \le u_1(s_1,k)$. Since s_1 is least preferred by player 1 when player 2 plays h, we also have $u_1(s_1,h) \le u_1(s_i,h)$. By case (1) we also have $u_2(s_1,k) < u(s_i,h)$. Thus we cannot also have $u_2(s_i,h) \le u_2(s_i,k)$, as in that case the subgame $\{s_1,s_i\} \times \{h,k\}$ would be a weak form of MP. Thus for any such s_i, $u_2(s_i,k) < u_2(s_i,h)$. Figure 2 visually summarises this argument. In particular, this implies that $u_2(s_n,k) < u_2(s_n,h)$.

Now let s_j be any strategy with $u_1(s_n,k) \le u_1(s_j,k)$. Since s_n is most preferred by player 1 when player 2 plays h, we also have $u_1(s_j,h) \le u_1(s_n,h)$. By the above, we also have $u_2(s_n,k) < u_2(s_n,h)$. Thus we cannot also have $u_2(s_j,h) \le u_2(s_j,k)$, as $\{s_n,s_j\} \times \{h,k\}$ would be a weak form of CO. Thus for any such s_j, $u_2(s_j,k) > u_2(s_j,h)$. Figure 3 visually summarises this argument.

However, we have now discussed all strategies $s_i \in S_1$, and in each case $u_2(s_i,k) < u_2(s_i,h)$, and so we find that strategy h dominates strategy k for player 2, contradicting our original assumption. Case (2) follows identical reasoning, swapping CO and MP and concluding with k dominating h. □

Corollary 4.2. Every strategy in a non-dominance-solvable preference-zero-sum game takes part in an MP subgame. Likewise, every strategy in a non-dominance-solvable preference-potential game takes part in an CO subgame.

Proof. By Theorem 4.1, every strategy surviving iterated dominance in a game takes part in a subgame which is a weak form of CO or MP. If the game is preference-zero-sum, then every CO subgame is also a weak form of MP, and likewise in a preference-potential game every MP subgame is also a weak form of CO, proving the result. □

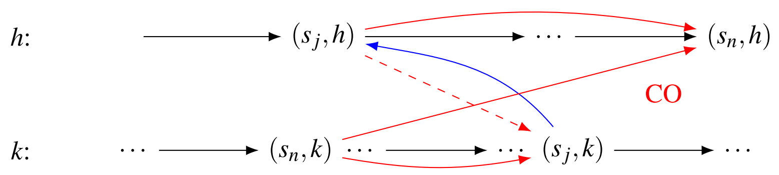

Figure 3. For s_j where (s_j,k) succeeds (s_n,k), the arc $(s_j,k) \longrightarrow s_j,h)$ must go from k to h (blue); if instead the arc goes from h to k (red, dashed) the subgame $\{s_j,s_n\} \times \{h,k\}$ is CO.

Corollary 4.3 (Zero-Sum–Potential–Dominance Theorem)**.** Any generic game that is both preference-zero-sum and preference-potential is dominance-solvable.

Proof. By Corollary 3.7, generic preference-zero-sum games do not contain CO, and generic preference-potential games do not contain MP, but games containing neither are dominance-solvable by Theorem 4.3. □

References

1. Bang-Jensen, J. & Gutin, G. Z. *Digraphs: theory, algorithms and applications* (Springer Science & Business Media, 2008).
2. Myerson, R. B. *Game theory: analysis of conflict* (Harvard university press, 1997).
3. Candogan, O., Menache, I., Ozdaglar, A. & Parrilo, P. A. Flows and decompositions of games: Harmonic and potential games. *Math. Oper. Res.* **36**, 474–503 (2011).
4. Papadimitriou, C. & Piliouras, G. Game dynamics as the meaning of a game. *ACM SIGecom Exch.* **16**, 53–63 (2019).
5. Omidshafiei, S. *et al.* α-rank: Multi-agent evaluation by evolution. *Sci. reports* **9**, 1–29 (2019).
6. Fudenberg, D. & Tirole, J. *Game Theory* (MIT press, 1991).
7. Morris, S. & Ui, T. Best response equivalence. *Games Econ. Behav.* **49**, 260–287 (2004).
8. Hwang, S.-H. & Rey-Bellet, L. Strategic decompositions of normal form games: Zero-sum games and potential games. *Games Econ. Behav.* **122**, 370–390 (2020).
9. Hwang, S.-H. & Rey-Bellet, L. Simple characterizations of potential games and zero-sum equivalent games. *J. Econ. Theory Econom.* **31**, 1–13 (2020).
10. Candogan, O., Ozdaglar, A. & Parrilo, P. A. Dynamics in near-potential games. *Games Econ. Behav.* **82**, 66–90 (2013).
11. Hammack, R. H., Imrich, W. & Klavžar, S. *Handbook of product graphs*, vol. 2 (CRC press, 2011).
12. Monderer, D. & Shapley, L. S. Potential games. *Games economic behavior* **14**, 124–143 (1996).
